# “Antibiotics are for everyone, our past and our future generations, right? If antibiotics are dead, we will be in big trouble”: Building on community values for public engagement on appropriate use of antibiotics in Singapore

**DOI:** 10.3389/fpubh.2022.1001282

**Published:** 2022-09-30

**Authors:** Huiling Guo, Zoe Jane-Lara Hildon, Angela Chow

**Affiliations:** ^1^Department of Preventive and Population Medicine, Office of Clinical Epidemiology, Analytics, and Knowledge, Tan Tock Seng Hospital, Singapore, Singapore; ^2^Saw Swee Hock School of Public Health and National University Health System, National University of Singapore, Singapore, Singapore; ^3^National Centre for Infectious Diseases, Ministry of Health, Singapore, Singapore; ^4^Lee Kong Chian School of Medicine, Nanyang Technological University, Singapore, Singapore

**Keywords:** antimicrobial resistance, shared decision-making, trusting relationships, continuity of care, community values, public engagement

## Abstract

**Introduction:**

Shared decision-making (SDM) and trust building through continuity of care are known to play a pivotal role in improving appropriate antibiotic prescribing and use.

**Problem:**

However, less is known about *how* to effectively leverage these factors when present—or overcome them when not—to address community needs and improve patient liaison.

**Methods:**

We addressed this question using a convergent parallel mixed-methods design. Focus group discussions (*N* = 13; August 2018–September 2020), were analyzed alongside a nationally-representative cross-sectional survey (*N* = 2004; November 2020–January 2021), in Singapore. Descriptive quantitative analyses and multivariable logistic regression were undertaken to examine antibiotic knowledge and factors associated with preference for SDM. Qualitative applied thematic analysis was integrated with these data to further explain the findings.

**Findings:**

Poor knowledge and misbeliefs on appropriate antibiotic use and antimicrobial resistance (AMR) were identified. For example, only 9% of the surveyed population understood that AMR occurs when the bacteria, not the human body, become resistant to antibiotics. Qualitative data corroborated the survey findings and suggested a shared value was placed on public education to avoid the fallout from resistant bacterial strains on current and future generations. This study also identified the opportunity to harness community trust in primary care doctors, who were described as highly valued educators for antibiotic use and AMR. Those who had trust in doctors were 75% more likely to prefer SDM (aOR 1.75, 95% CI 1.10–2.77, *P* = 0.017), especially adults aged ≥50 years who were receiving continued care with a regular doctor (aOR 1.83, 95% CI 1.18–2.86, *P* = 0.007). Continuity of care was observed to value-add SDM by building trusting relationships, though it was often absent in younger populations.

**Conclusion:**

This study highlights the long-term value-add of building on cultural capital pertaining to appropriate antibiotic use and AMR, by leveraging on the role of trust in doctors, desire for SDM and anchoring these in continuity of care when possible.

**Recommendations:**

Using focused messaging and exploring alternative channels of communications such as annual check-ins or tele-consultations with a regular doctor, and emphasizing continuity of care across all age groups would help bridge the identified gaps.

## Introduction

Global annual mortality attributable to antimicrobial resistance (AMR) was projected to reach 10 million by 2050 (1). This estimated number is comparable with the annual global excess death count of an average of 7.5 million reported for the coronavirus disease 2019 (COVID-19) in the first 2 years of the pandemic (2), suggesting an urgent need to slow down AMR progression before it becomes the next pandemic.

Overprescribing of antibiotics is one of the major causes of AMR (3). Reasons include patient demands, clinical uncertainties, fear of missing diagnosis, and fear of medico-legal issues (4–10). However, one-sided information delivery through educational materials focused on encouraging doctors to improve appropriate antibiotic prescribing and nationwide campaigns to raise public awareness on AMR have limited effects (11–13). In contrast, systematic reviews have shown that shared decision-making (SDM) between patients and doctors enables better chances of reducing inappropriate antibiotic prescribing and use (11, 14, 15). Furthermore, the process of SDM is known to be buoyed up by receiving continuity of care from a regular doctor, and having mutual trust (7, 16–18). The importance of these relationships has been explored in a qualitative study conducted amongst primary care doctors practicing in Singapore, which has positioned these constructs at the core of a VALUE model of SDM for antibiotic prescribing (18).

The model highlights the importance of starting with—building up when lacking or drawing on when present—the doctor's *own* values and organizational culture to adhere to recommended practice and optimal patient care. Nevertheless, not every context will present the opportunity to influence or leverage such values. Continuity of care is not always possible, and trust takes time. In some cases, trust may be hard to win, if ever. To better navigate such scenarios, the central role of patients in navigating decisions around antibiotic use and adherence needs to be better understood. So far, existing literature indicates that the public's perceptions of SDM have been less explored, in favor of appraising satisfaction with clinical consultations post-SDM (11, 14).

Accordingly, the current study aims to better understand how to support the VALUE model's application in the primary care setting by accounting for the community's perspective and how to improve patient liaison around recommended antibiotic practices. We use a mixed-methods design informed by social and behavior change communication (SBCC) traditions (19) to firstly, assess gaps in knowledge, as well as intentions and behavioral follow-through to inform related ***messaging*** needs. Next, we examine for whom trust in doctors, continuity of care and SDM are valued to inform ***targeting*** for practitioner-led intervention design. Lastly, we explore the role of trust, how it is established and in particular how trusted sources can be leveraged *via* ***multiple channels*** to share information.

Our study defines SDM, following Elwyn et al. as a three-step process: (a) providing reasonable options to patients, (b) using decision aids to describe these, and (c) exploring patient preferences and making choices together with the doctor (20). The planned analysis acknowledges these steps, starting with defining specific knowledge and intentions or behavioral gaps that help to define how “reasonable options” to use antibiotics appropriately should be messaged and communicated. In addition, we opted to dig deeper on understanding how to target these decision aids, building on a previous study conducted in Singapore, which highlighted that poor knowledge of antibiotic use and AMR in younger age groups drives larger extents of inappropriate antibiotic practices (21). Lastly, best channels through which related information may be strategically used are assessed. Existing channels and campaigns in our present context are discussed below.

Overall, these analyses will help us identify areas for theory-informed intervention design and strategic implementation to improve antibiotic use in the primary care setting, *via* SDM processes, adding to what is already know from the practitioner's perspective, based on the existing identified VALUE-driven model (18).

## Methods

### Mixed-methods study design

This is a convergent parallel mixed-methods study. A nationally-representative community-based survey was conducted (November 2020–January 2021) on a randomly selected sample of Singapore residents (citizens and permanent residents) aged 21 years and above. The sampling frame and data collection methods are fully reported elsewhere (21). Separately, 13 focus group discussions (FGDs) were conducted (August 2018–September 2020). The Standards for Reporting Qualitative Research (SRQR) (22) was used to report qualitative methods, and quantitative procedures were integrated within.

All study methods and procedures were reviewed and approved by the National Healthcare Group Domain Specific Review Board of Singapore (Reference Number: 2017/01179).

### Singapore context

The survey was conducted during the COVID-19 pandemic (November 2020–January 2021), after a national lockdown was lifted. Working adults, who were previously office-based, remained mostly in a “work-from-home” mode. Majority of students enrolled in higher learning institutes were attending classes online. On the other hand, FGD recruitment was disrupted by the COVID-19 pandemic (January 2020–August 2020) due to the early stages of national containment of community COVID-19 transmission. FGDs were resumed and completed in September 2020 with strict compliance to the nation's safe management measures.

Between 2018 and 2020, the annual AMR campaign message by the Singapore Health Promotion Board was “Fighting the flu virus is not my battle. Talk to your doctor for the treatment you need” (23). It was intensively disseminated through posters at public areas (bus stops, rapid transit system stations), brochures, tissue packs, television advertisements, social media posts and YouTube advertisements during the annual World Antibiotics Awareness Week in November.

### Quantitative component

#### Survey instrument and variable selection

The survey questionnaire addressed antibiotic use and AMR. These included questions on knowledge, trust in information sources and doctors, as well as continuity of care, which were selected for analysis.

Knowledge questions were presented as True/False/Don't know. Questions on attitude and trust in doctors were presented in a 5-point Likert scale (strongly disagree to strongly agree) and dichotomized in the manner described below. Trust in information sources for health-related matters or medicines was presented in a 5-point Likert scale (never to completely) and dichotomized into 2 categories: trust (moderately/a lot/completely) vs. do not trust (never/rarely). Additional demographic information was also collected.

The dependent variable was defined by the statement “I would want my doctor to discuss with me and make the decision on antibiotic prescribing with me” (24). Respondents who agreed to this statement (strongly agree/agree) would be categorized as *preferring SDM on antibiotic prescribing with their doctors*. The independent variable on patient-acquired, all-round *trust in doctors* was tabulated using a composite score. It was composed of a 9-statement scale developed by Hall et al. (25), and agreement (strongly agree/agree) to all 7 positive statements, and disagreement (neither agree or disagree/disagree/strongly disagree) to both negative statements.

Positive statements included: (1) doctors in general care about their patients' health just as much as their patients do, (2) doctors are extremely thorough and careful, (3) I completely trust doctors' decisions about which medical treatments are the best, (4) doctors are totally honest in telling their patients about all the different treatment options available for their conditions, (5) doctors think only about what is best for their patients, (6) doctors always use their very best skill and effort on their patients, and (7) I have no worries about putting my life in the hands of doctors.

Negative statements were: (1) sometimes doctors care more about what is convenient for them than about their patients' medical needs, and (2) sometimes doctors do not pay full attention to what patients are trying to tell them.

Lastly, another independent variable on *continuity of care* was defined as reportedly seeking medical attention from a regular doctor.

#### Quantitative data analysis

Proportions were tabulated and chi-squared test was used to compare differences between them. Multivariable logistic regression was then performed to determine the independent factors associated with preference for SDM on antibiotic prescribing with doctors. Covariates were selected through assessing the Akaike information criteria, Bayesian information criteria and likelihood ratios, and included in the final regression model to adjust for potential confounding. Interactions between covariates were individually explored and product terms were also included in the final model. Effect measure modification due to socio-demographic factors was further assessed. Statistical significance was defined as *P*-value < 0.05. Statistical analyses were conducted in Stata version 14.0 (StataCorp LLC, College Station, Texas US).

### Qualitative component

#### Researcher team composition and reflexivity

A semi-structured topic guide (Supplementary File Annex 1) was developed by HG (Female, MPH, Research Fellow) based on previous findings from the literature (26–32). Pilot interviews were conducted with co-workers of varying educational levels and with no prior medical knowledge to ensure content validity and proper phrasing of questions. Three research assistants, all females, bilingual graduates and trained in qualitative fieldwork, facilitated or took notes for the FGDs in the preferred language of the participants (English, Mandarin, Malay or Tamil).

#### Focus group discussions (FGDs) sampling and data collection

Invitation letters were disseminated to the community through community networks or recruitment drives. Interested members of the community left their contact details with the study team and were later contacted *via* email or telephone. Informed consent and basic demographic details were collected on the day of the FGD. Each FGD lasted for 90 mins. The topic guide consisted of questions pertaining to knowledge, attitudes and perceptions toward antibiotic use and AMR, antibiotic practices and also interactions with primary care doctors on the use of antibiotics.

#### Units of study

Singapore residents (citizens and permanent residents) aged 21 years and above were purposively sampled with maximum variation to ensure representation from different ethnic (Chinese, Malay and Indian) and age (21–49 years old and ≥50 years old) groups. A good mix of education level was also considered. To reach data and meaning saturation (33), at least two focus groups were required per stratum (i.e., older Chinese, younger Chinese, older Malay, younger Malay, older Indian and younger Indian). Hence, in this study, a minimum total number of 12 focus groups was planned. All potential participants were screened and included in the study if they were able to answer the question “Do you know what are antibiotics?.”

#### Qualitative data processing and analysis

Each FGD was audio-recorded and data were transcribed verbatim. Applied thematic analysis was undertaken (34). Steps included data familiarization, segmenting the data according to topics pertinent to the current study objectives, and agreeing on a coding framework, as well as describing emergent themes. The coding framework was guided by identification of elements of VALUE model for antibiotic prescribing in the primary care setting (18). These included knowledge and understanding of antibiotic use and AMR, the presence and role of continuity of care, trusting patient-doctor relationship and active liaison with patients that lead to SDM processes on antibiotic prescribing. ATLAS.ti 9 was used to manage the qualitative data and record emergent themes.

#### Techniques to enhance trustworthiness of qualitative analysis

Regular meetings were conducted with a senior member of the team. Emergent themes and sub-themes were discussed and a consensus reached on the meaning of the data. Saturation was judged to have been achieved at over two-thirds of the way through the coding process.

### Mixed methods reporting

Both datasets were analyzed separately and integrated at the reporting stage as appropriate. Qualitative main themes are reported by highlighting these in ***bold-italics***, while supporting themes are narrated alongside these. Each objective is addressed in turn.

## Results

Out of 4791 households approached, 2004 (41.8%) respondents took part in the survey. They were representative of the Singapore population in 2020 (35), and most sought antibiotics from a GP and had a regular doctor (Supplementary Tables S1, S2). Thirteen FGDs were conducted, with a good distribution of ethnic groups and balance of education level, diversity of gender and ages was also achieved (Supplementary Table S3).

### Informing messaging needs to improve knowledge of antibiotic use and antimicrobial resistance (AMR)

**Figure 1:**
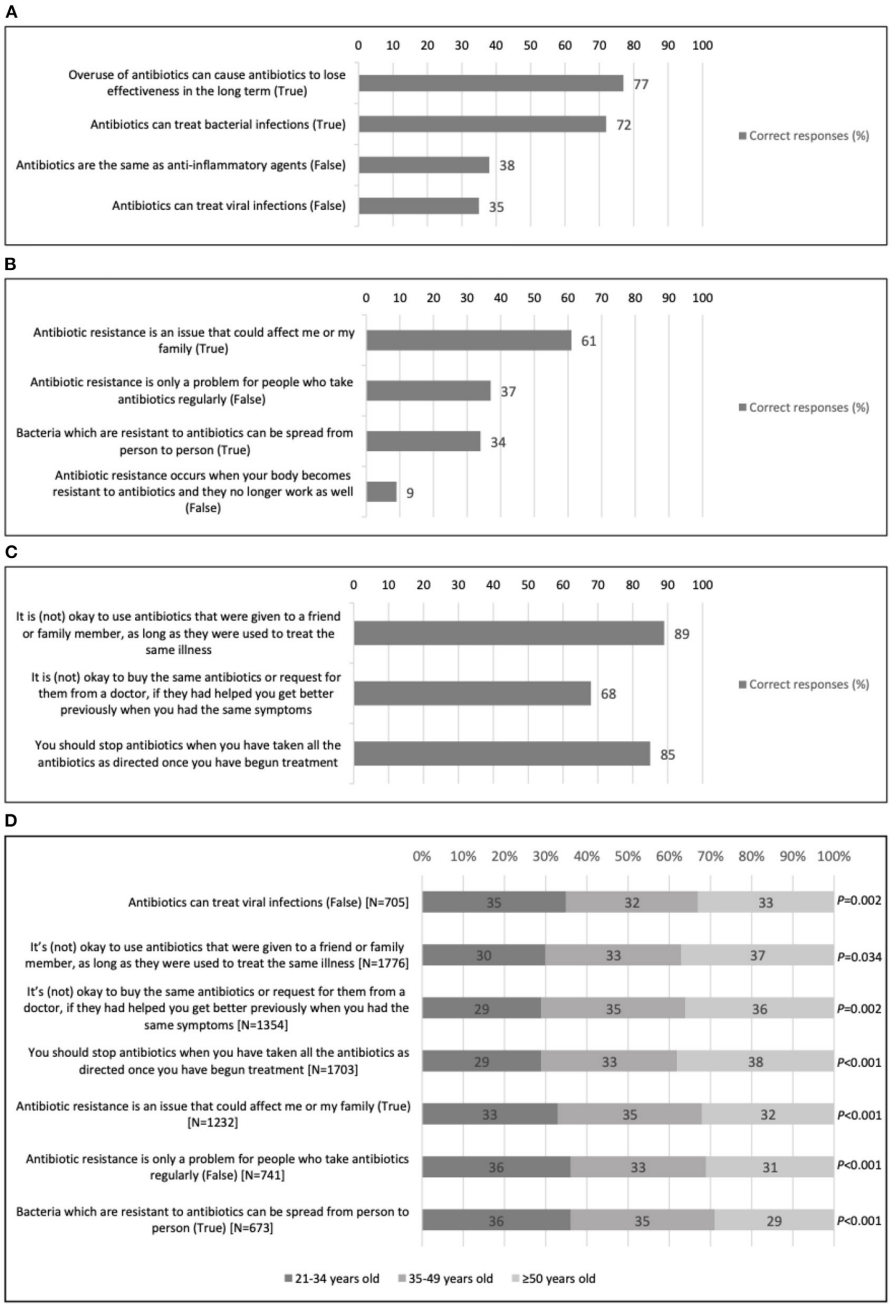
Proportion of correct responses from 2004 Singapore residents on statements pertaining to antibiotic use and antimicrobial resistance (AMR), surveyed between November 2020 and January 2021. **(A)** Knowledge of statements related to understanding how antibiotics work. **(B)** Knowledge of statements related to antimicrobial resistance (AMR). **(C)** Knowledge related to appropriate ways of obtaining and taking antibiotics. **(D)** Understanding differences in correct response in **(A–C)**, stratified by age (only significant trends reported).

Descriptive quantitative analyses are summarized in Figures 1A–D. These findings are integrated with qualitative thematic analyses, summarized in Tables 1, 2 with illustrative quotes.

#### Gaps in knowledge of antibiotic use and antimicrobial resistance (AMR)

Quantitative data showed a good understanding surrounding the need for using antibiotics cautiously and intentionally (Figure 1A). For instance, a large proportion knew that overuse of antibiotics can cause them to lose effectiveness in the long term (77%). It was also largely known that antibiotics are for the treatment of bacterial infection (72%), even though other misconceptions existed. These misconceptions centered on far fewer recognizing the falsehood that antibiotics could be used to treat viral infection (35%) and believing that antibiotics had anti-inflammatory properties (38%).

Qualitatively, see Table 1A, we identified ***specific gaps in the community's knowledge*** ***around reasons to take and minimize antibiotic use where appropriate***, which corroborated with the above.

**Table 1A:** Gaps in knowledge, themes collated from focus group discussion data.

**Themes**	**Sub-themes**	**Illustrative quotes**
Specific gaps in the community's knowledge around reasons to take and minimize antibiotic use where appropriate	• Inability to differentiate between bacteria and viruses	“Antibiotics [are] for virus…if you're not in a medical line, you [will] get confused with bacteria, virus, germs…but I know that antibiotics are for viruses.” -FGD11, Indian, 35–49 years old
	• Misconception that an antibiotic is the same as a painkiller, or an anti-inflammatory agent	“They said it was to reduce inflammation…Ya, for disinfection…Only take it when the illness is severe.” -FGD03, Chinese, ≥50 years old
	• Overuse of antibiotics was seen as leading to the “body building immunity” against the antibiotics, not the bacteria itself becoming resistant	“If doctor gives you medications, once you are well…once you are healed, stop. If not, the next time you are sick…It's like body has become used to it…the immunity toward antibiotics is inside of us…our protection is no longer there. So even if we eat antibiotic, it would no longer be effective.” - FGD02, Malay, ≥50 years old
Poor understanding of AMR	• Lay beliefs rather than scientific consensus were commonly being used to define the term “antibiotic resistance”	“Is it something where your body doesn't work on the antibiotics, already reached its maximum potency, like a dependency…It's like reached its limit…won't work for you anymore, is that it?” - FGD12, Indian, ≥50 years old
	• Misconception that effects of antibiotic resistance are cumulative by age	“So we are the pioneer. We eat more, we take more of this [referring to antibiotics]. When it comes to resistance…it is possible that is not very effective to the elderly. Because we already built up something inside [our body] already.” - FGD04, Chinese, 35–49 and ≥50 years old

These included the explicit inability to differentiate between bacteria and viruses. Also, the misconception that an antibiotic is the same as a painkiller, or an anti-inflammatory agent. Furthermore, the underlying rationale as to why the overuse of antibiotics would interfere with their effectiveness in the long term was misunderstood: it was seen as leading to the “body building immunity” against the antibiotics, not the bacteria itself becoming resistant. Relatedly, one participant also had the erroneous thought that antibiotics are taken to strengthen one's immune system. These beliefs had influence over how antibiotics were taken, as explained below.

In general, the term “antibiotic resistance” was incomprehensible to the community (Figure 1B). Echoing previously shared qualitative findings, only 9% of the respondents realized that it is erroneous to believe that AMR occurs when the body becomes resistant to antibiotics. Relatedly, just 34% were aware that bacteria which are resistant to antibiotics can spread from person to person. Overarchingly, qualitative findings showed a ***poor*** ***understanding of AMR*** and indicated that lay beliefs rather than scientific consensus were commonly being used to define the term “antibiotic resistance.”

The term was being used to infer mechanisms that only affect those who overdose on or overuse antibiotics, and therefore would need even more antibiotics to achieve an effect or the body itself had developed resistance to the antibiotic. Some expressed that the effects of antibiotic resistance were cumulative by age, since older adults would have taken more of such medications over their life time. Such rationales may explain why 61% of survey respondents reported that antibiotic resistance was an issue that may affect them or their families, despite most not understanding the mechanism by which this occurred.

#### Intentions and behavioral follow-through on antibiotic use

Most respondents in the survey reported that they understood that antibiotics should not be shared with others (89%) and that they should be taken as directed (85%) (Figure 1C). Despite these quantitative results, focus group participants commonly shared that it was not unusual for them or their family members to not finish a full course of antibiotics once they started to feel better. Qualitative data highlights ***while the best ways to obtain antibiotics and*** ***the advice on taking them was generally known, this did not always translate to good*** ***practices*** (Table 1B). The misconception that the body, not the bacteria, became resistant was one reason why the full dose of prescribed antibiotics might not be completed.

**Table 1B:** Intentions and behavioral follow-through, themes collated from focus group discussion data.

**Themes**	**Sub-themes**	**Illustrative quotes**
While the best ways to obtain antibiotics and the advice on taking them was generally known, this did not always translate to good practices	• The misconception that the body, not the bacteria, became resistant was one reason why the full dose of prescribed antibiotics might not be completed	“I try not to finish in a way I got my body to be used to it [referring to antibiotics].” - FGD08, Chinese, 21–34 years old
	• Requesting tried-and-tested antibiotics was driven by the desire to recover from an illness faster	“Because I wanted to recover faster. I had some event [going] on, so I requested them because antibiotics normally works much faster. So I did request.” - FGD10, Indian, 21–34 years old

In addition, 68% of survey respondents correctly responded that it would not be advisable to buy the same antibiotics or request them from doctors simply because these had helped with similar symptoms previously (Figure 1C); while focus group participants often described such a request as being reasonable because it was driven by the need to recover from an illness faster. There were small differences by age in knowledge (Figure 1D); in general, those aged 35 years and older were more apt at answering correctly on the appropriate ways of obtaining antibiotics while younger people had marginally better understanding of statements relating to AMR.

### Targeted approaches stratified by age for improving public knowledge and appropriate antibiotic use

**Figure 2:**
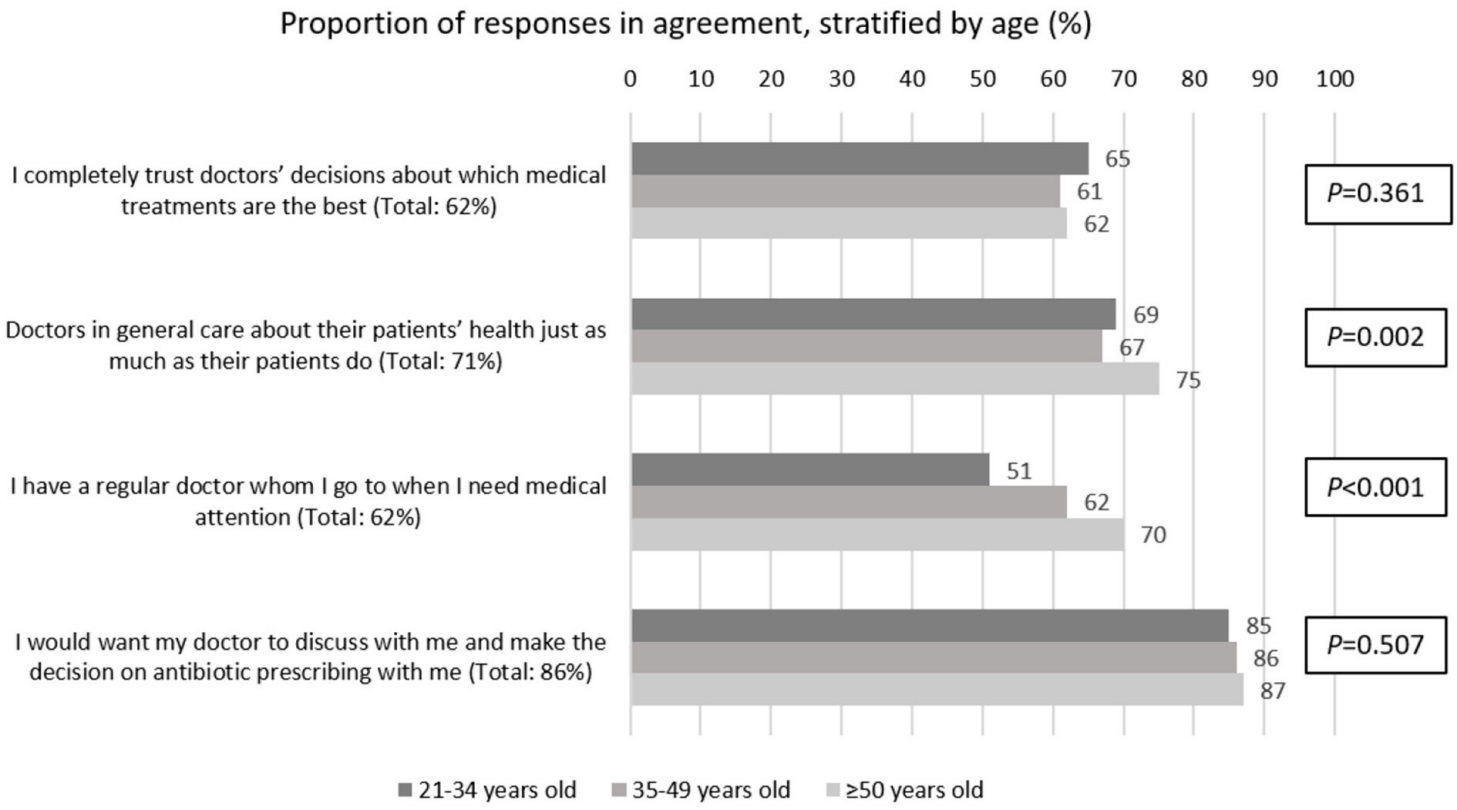
Proportion of responses from 2004 Singapore residents, surveyed between November 2020 and January 2021, agreeing to these statements pertaining to trust in doctors, continuity of care and shared decision-making (SDM) for antibiotic prescribing, stratified by age.

Quantitative descriptive results are collated in Figure 2 and regression analyses are summarized in Tables 2A,B. Analyses are presented by stratifying age, and they also built on existing findings which have shown that poor knowledge of antibiotic use and AMR and inappropriate antibiotic use in the general population are modified by age; with younger adults being less informed and likely to have worse outcomes (21).

**Table 2A:** Univariate and multivariable logistic regression examining factors associated with preference for shared decision-making on antibiotic prescribing, *N* = 2004.

**Variable**	**Do not prefer SDM** ** (*N* = 280)**	**Prefers SDM** ** (*N* = 1,724)**	***P*-value**	**Univariate analysis**			**Model 1: Without interaction terms**			**Model 2: including interaction terms**		
				**Unadj. OR**	**95% CI**	***P*-value**	**Adj. OR**	**95% CI**	***P*-value**	**Adj. OR**	**95% CI**	***P*-value**
**Trust in doctors**, ***N*** **(%)**												
Yes	22 (8)	227 (13)	**0.012**	1.79	1.13–2.81	**0.014**	1.75	1.10–2.77	**0.017**	1.75	1.10–2.77	**0.017**
**Has continuity of care with a regular doctor**, ***N*** **(%)**												
Yes	156 (56)	1,077 (62)	**0.031**	1.32	1.03–1.71	**0.031**	1.27	0.98–1.65	0.075	0.93	0.60–1.45	0.746
**Gender**, ***N*** **(%)**												
Male	132 (47)	822 (48)	0.867	1.02	0.79–1.32	0.867	1.02	0.79–1.32	0.860	1.02	0.79–1.31	0.907
**Age group**, ***N*** **(%)**												
21–34 years old	94 (34)	521 (30)	0.507	Ref	–	–	Ref	–	–	Ref	–	–
35–49 years old	90 (32)	568 (33)		1.14	0.83–1.56	0.415	1.18	0.86–1.62	0.317	1.05	0.65–1.67	0.852
≥50 years old	96 (34)	635 (37)		1.19	0.88–1.62	0.259	1.37	0.97–1.93	0.075	0.94	0.58–1.52	0.796
**Ethnic group, N(%)**												
Non-Chinese	61 (22)	505 (29)	**0.010**	1.49	1.10–2.01	**0.010**	1.59	1.17–2.17	**0.003**	1.60	1.18–2.19	**0.003**
**Highest educational level, N(%)**												
Lower educated (Post-secondary & below)	105 (37)	591 (34)	0.294	Ref	–	–	Ref	–	–	Ref	–	–
Higher educated (Diploma & above)	175 (63)	1,133 (66)		1.15	0.89–1.49	0.294	1.36	1.01–1.82	**0.042**	1.38	1.03–1.85	**0.033**
**Has ever had at least 1 chronic illness**, ***N*** **(%)**												
No	188 (67)	1,168 (68)	0.840	1.03	0.79–1.35	0.840	–	–	–	–	–	–
**Family member/friend working in healthcare sector**, ***N*** **(%)**												
Yes	133 (47)	943 (55)	**0.025**	1.33	1.04–1.72	**0.025**	–	–	–	–	–	–
**Interaction between continuity of care and being 35**–**49 years old**												
Product term	–	–	–	–	–	–	–	–	–	1.29	0.68–2.44	0.425
**Interaction between continuity of care and being** **≥50 years old**												
Product term	–	–	–	–	–	–	–	–	–	1.97	1.05–3.67	**0.034**

Bolded values indicate statistical significance of P < 0.05.

**Table 2B:** Association between preference for shared decision-making on antibiotic prescribing and continuity of care, according to age group, *N* = 2004.

**Preference for SDM**	**21**–**34 years old**		**35**–**49 years old**			≥**50 years old**		
	**(*****N*** = **615)**		**(*****N*** = **658)**			**(*****N*** = **731)**		
	**OR**	**95% CI**	**OR**	**95% CI**	***P*-interaction^a^**	**OR**	**95% CI**	***P*-interaction^a^**
**Unadjusted analysis**								
Lacks continuity of care	Ref	–	Ref	–	0.374	Ref	–	**0.007**
With continuity of care	0.98	0.63–1.53	1.23	0.78–1.93		1.83	1.18–2.85	
**Adjusted analysis** ^**b**^								
Lacks continuity of care	Ref	–	Ref	–	0.425	Ref	–	**0.007**
With continuity of care	0.93	0.60–1.45	1.20	0.76–1.89		1.83	1.18–2.86	

a Multiplicative scale.

b Adjusted for trust in doctor, gender, ethnic group, and highest educational level.

Bolded values indicate statistical significance of P < 0.05.

#### Trust, continuity of care, and shared decision-making (SDM)

Overall, in the present study, only a small majority of 62% reported having complete trust in doctors' decisions about which medical treatments are the best (Figure 2). Descriptive data showed a small effect of age relating to general trust in doctors, with those aged ≥50 years being most likely to believe that doctors in general care about their patients' health just as much as their patients do (21–34 years old: 69%; 35–49 years old: 67%; ≥50 years old: 75%, *P* = 0.002). Similarly, amongst the 62% of respondents who had continuity of care with a regular doctor, there were larger proportions of older respondents who medically attended with a regular doctor (21–34 years old: 51%; 35–49 years old: 62%; ≥50 years old: 70%, *P* < 0.001). In contrast, quite a few more (86%) reported a preference for SDM but there was no statistically significant difference between age groups on such preference (21–34 years old: 85%; 35–49 years old: 86%; ≥50 years old: 87%, *P* = 0.507).

Upon adjusting for potential confounders, respondents who scored as trusting their doctors based on the scale measuring all-round trust in doctors, as developed by Hall et al. (25), were 75% more likely to prefer SDM when seeking antibiotic prescriptions (aOR 1.75, 95% CI 1.10–2.77, *P* = 0.017) (Table 2A). Though there was no significant effect from continuity of care on preference for SDM after adjusting for potential confounders, there was a significant multiplicative effect of age on these associations (Table 2B). In those aged 50 years and above, it was found that when seeking antibiotics, those with continuity of care were 83% more likely to prefer SDM with their doctors, compared to those without it (aOR 1.83, 95% CI 1.18–2.86, *P* = 0.007).

### Trust building and the use of multiple communication channels to promote education on appropriate antibiotic use and antimicrobial resistance (AMR)

**Figure 3:**
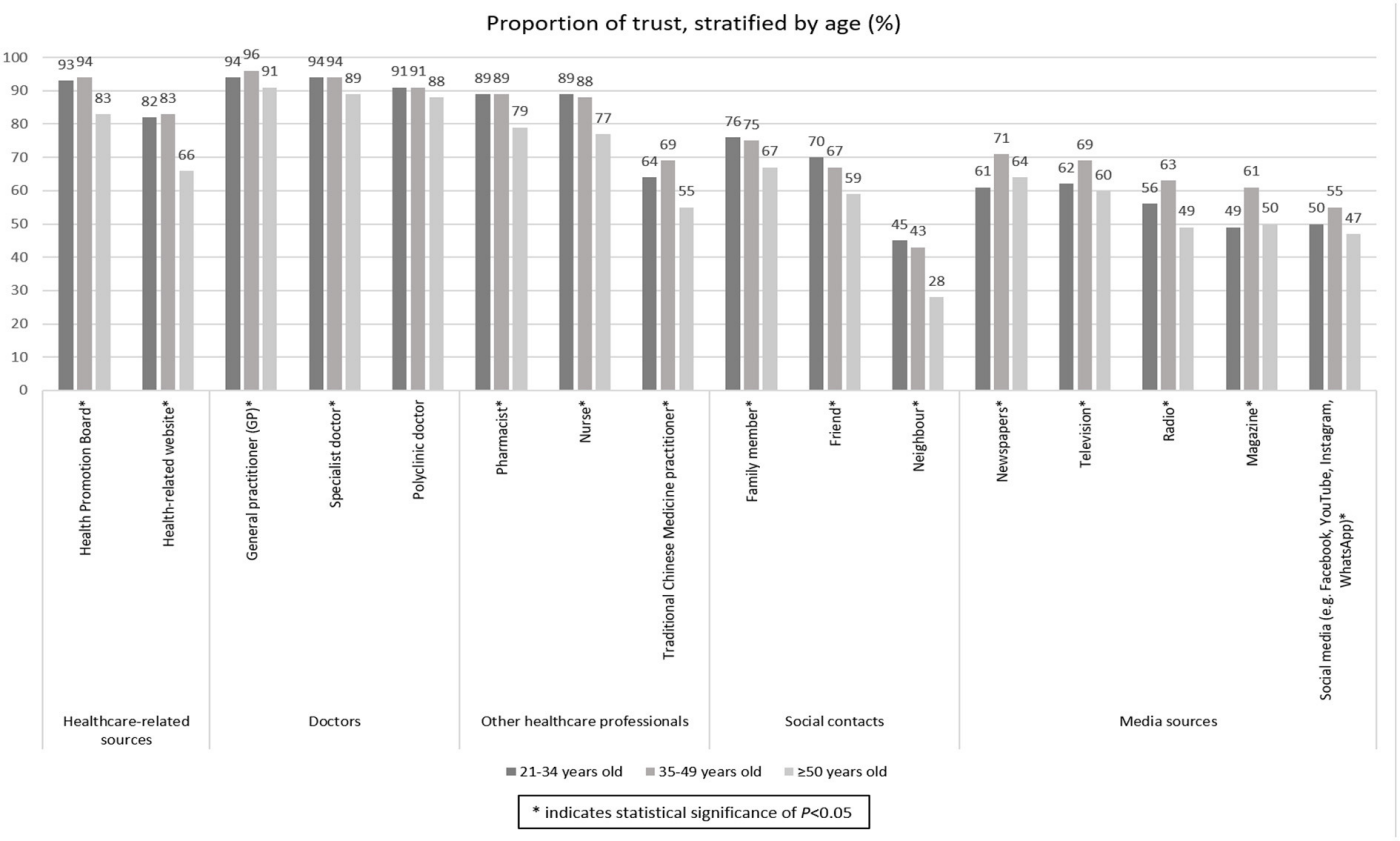
Proportion of trust on different health information sources of 2004 Singapore residents surveyed between November 2020 and January 2021, stratified by age. The * was used to denote categories with statistical significance.

Descriptive quantitative analyses stratified by age are listed in Figure 3. These findings are supplemented with qualitative thematic analysis, summarized with illustrative quotes in Tables 3A–C.

**Table 3A:** Trust building, themes collated from focus group discussion data.

**Themes**	**Sub-themes**	**Illustrative quotes**
Value-add through taking time to build trust	• In some cases, trust was not a given	“Too much false information out there. People no longer trust already. Even doctors, not a lot of people trust [them]a” - FGD08, Chinese, 21–34 years old
	• Trust can be built by better communication and sharing of knowledge	“Because the doctor also did not inform us of anything. “You just eat this medication” like this…or breakdown what will happen…the doctor didn't let us know. Just asked us to finish eating this [referring to antibiotics], that's all we know.” • FGD05, Chinese, ≥50 years old
Valuing of public education on appropriate antibiotic use and AMR	• Importance of twinning trusted role of doctor with outreach and scientific information	“For the general public, usually whatever instruction is given by the doctor, they [follow]. Because these are the doctor's instructions. But it's not being widely published in the newspapers, so we don't know enough [to understand why instructions are given as they are].” - FGD02, Malay, ≥50 years old “Everybody, should know about this issue, because antibiotics are for everyone, for our past and our future generations, right? If antibiotics are dead, we will be in big trouble.” FGD04, Chinese, 35–49 yo and ≥50 years old
	• Observed lack of public outreach on AMR as compared to other chronic or lifestyle diseases, e.g., diabetes	“Most of the common people, the public, most of us, we are not alerted of this antibiotic resistance. We are not alerted, you see. So we don't know what [is the] cause, what is the outcome of it, the seriousness is that when you get antibiotic resistance.” - FGD06, Malay, ≥50 years old “There were a lot of campaigns and there was a lot of awareness built around diabetes because it's a serious issue that we are handling. And just seeing it [referring to campaign messages] again and again and again, it's always at the back of your mind.” - FGD10, Indian, 21–34 years old

**Table 3B:** Shared decision-making, themes collated from focus group discussion data.

**Theme**	**Sub-themes**	**Illustrative quotes**
Valuing of SDM on antibiotic prescribing	• Expressed by the desire to have healthcare professionals as main focal point of education	“If you do too much mass education…it's meaningless to me. I don't know what is antibiotic because I don't take antibiotic, right? Unless I am sick and I need to take antibiotic, and the person who prescribes it to me or at the pharmacy tells me “You must make sure you finish this for this reason.” That education will be very helpful. And maybe at the same time, give me a pamphlet. That way I will read and say, “Okay, I know why I need to complete.”” • FGD05, Chinese, ≥50 years old
	• Initiation of SDM was not experienced as a “matter of course”—it may, or may not happen	“From my personal experience, the doctor has never discussed it [referring to antibiotic prescribing] with me. And I think I would prefer that…perhaps, more doctors could discuss it with the patients.” - FGD10, Indian, 21–34 years old “The doctor said, “I want to prescribe this medicine [referring to antibiotics]. What are your opinions.”” - FGD09, Malay, 21–34 years old
	• Communication to redress the lack of SDM, for instance tackling poor knowledge and empowering patients, perceived as the doctor's responsibility	“From the point where the medicine is being prescribed…say “Okay, I'm going to give you this. Do you understand what you are taking? Do you understand the risk behind taking it, and properly taking it and what not properly taking it would do?” And then once you finish, sign it…you should make it mandatory for all GPs and healthcare providers.” - FGD03, Chinese, ≥50 years old

**Table 3C:** Continuity of care, themes collated from focus group discussion data.

**Theme**	**Sub-themes**	**Illustrative quotes**
**Valuing of continuity of care**	• Returning for follow-up consultations was directly connected to valuing existing relationships	“I always go and see the same doctor. Never [do I] go to other clinics… [if] my condition still did not improve, he will give me antibiotics.” - FGD02, Malay, ≥50 years old
	• Also, enabled or hindered by practical factors, such as proximity, waiting times, speed and efficiency of diagnosis etc.	“You know, think about the doctors, the queues just put me off.” • FGD11, Indian, 35–49 years old “If every time you visit that doctor and you don't recover, if every time you need to consult twice or thrice, then stop going there on the next time.” -FGD01, Chinese, ≥50 years old

Though older people were shown to have better continuity of care and apparent interpersonal relationships with their doctors, trust in doctors was cross-cutting across age bands, see Figure 2, which shows no significant difference across age bands for having complete trust in doctors' decisions about medical treatments. Similarly, when asked about preferences for trusted sources to gain information on health-related matters or medicines (Figure 3), there were very small differences across age bands on preferences for doctors. Indeed, general practitioners (GPs) were reported as most trusted by all age bands compared to all other proposed channels of acquiring information. However, younger people were more receptive to information by other health professionals such as nurses, as well as social contacts such as friends and family, than those over 50 years of age.

Qualitatively, consensus across the age bands emerged pertaining to main themes such as the ***value-add through taking time to build trust*** (Table 3A) and enable SDM, as shown in Table 2A. It was shared that in some cases, trust between patients and doctors was not a given. Ways of doing this pivoted around better communication and sharing of knowledge. There was a clear demand for and expression toward ***valuing of public*** ***education on appropriate antibiotic use and AMR*. **The importance of twinning trusted role of doctor with outreach and scientific information dissemination was pointed out as a basis for protecting current and future generations from the risks of antibiotics being rendered ineffective. This was corroborated by an observed lack of public outreach on AMR as compared to other chronic or lifestyle diseases, e.g., diabetes.

***The valuing of SDM on antibiotic prescribing*** (Table 3B) was also notable by the expressed desire to have healthcare professionals as main focal point of education, supplemented by the use of other channels of communication and use of decision aids. In addition, it was shared throughout the focus groups that initiation of SDM was experienced as a “matter of course”—it may or may not happen. Interestingly, communication to redress this lack of SDM, for instance tackling poor knowledge and empowering patients, was perceived as the doctor's responsibility.

Lastly, the ***valuing of continuity of care*** (Table 3C) was directly connected to valuing existing relationships with ones' doctors. Continuity of care was also enabled or hindered by practical factors, such as proximity, waiting times, speed and efficiency of diagnosis etc. It was clear that continuity of care, though often preferred, may not always be possible. Models of primary care provision will need to account for such situations and enable ways of encouraging seeking and receiving medically sound advice when the potential need for antibiotics presents, despite these limitations.

## Discussion

This study provides important insights on what were the community's needs which should be addressed before and during SDM. It emphasizes the role of trust on educating patients to address their needs, promoting continuity of care and influencing their acceptance and desire for SDM with their doctors on antibiotic prescribing in primary care settings. The central role of trust in driving the community's preference for SDM with their doctors on antibiotic prescribing was evident, with those who trusted their doctors being far more likely to prefer SDM, as compared to those who did not trust their doctors.

SDM was not an unfamiliar concept within the community, with many wanting this to happen when seeking antibiotics and others sharing that primary care doctors were already practicing this. However, the community lacked empowerment to actively take part in SDM, despite their desires, due to a lack of medical knowledge, as self-perceived and as shown in current and previous quantitative findings (21). Being equipped with right information to make informed choices is key during SDM (20). There were obvious knowledge gaps of both antibiotic use and AMR, and presence of misbeliefs surrounding these topics amongst the community, which translated to inappropriate antibiotic use.

These study findings are reflective of existing literature (21, 36–38), though our qualitative findings further revealed that there could be reasonable intentions behind undesirable antibiotic practices. Our study also informs a model of how to build on the community's valuing of SDM and leverage the importance of following appropriate antibiotic behaviors to minimize the potential for AMR development and preserve present and future generations' access to effective antibiotic treatments. Desired antibiotic behaviors include seeking medical consultation before taking antibiotics, rather than demanding them; following doctor's advice on how to take them; and not sharing them with others or stocking them for future use unnecessarily.

**Figure 4:**
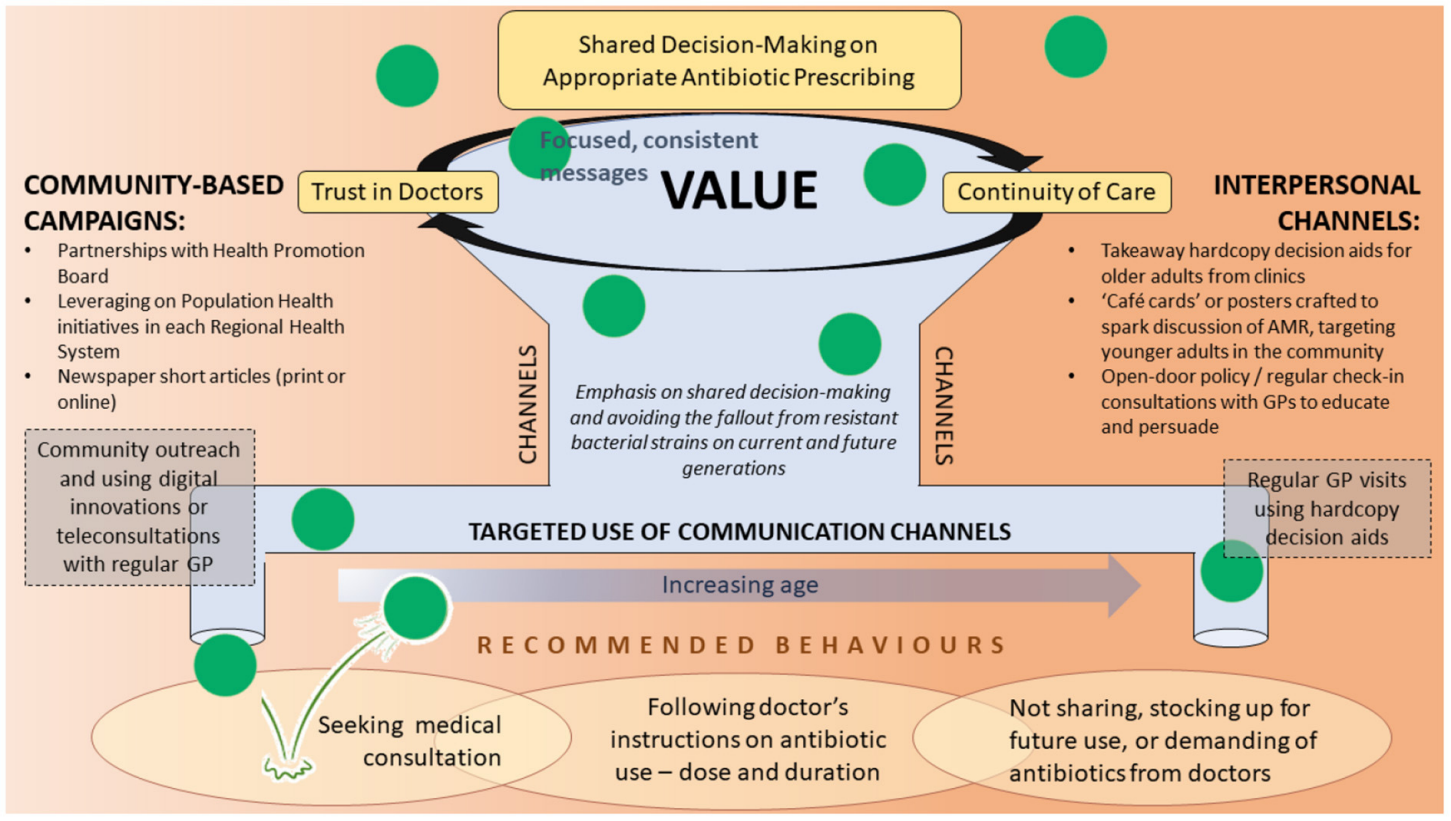
A strategic implementation model to guide the designing of interventions to improve appropriate antibiotic use in the general public.

The mixed-methods data informs a model of strategic planning by using cultural capital to value-add, and build on what is known, using: tailored message content design following the 7Cs of public health communications (39) (see Supplementary Table S4, for suggested message content); funneling these into desired behaviors using appropriate, age-segmented, targeted, multi-channel intervention aids extracted from our findings (Figure 4). Message content needs to be clear and consistent regardless of communication modality used and, we suggest, spearheaded or endorsed by the highly trusted medical professionals, especially GPs, partnering with the Health Promotion Board or the Regional Health Systems through community-based campaigns. Traditional modes of communication such as hardcopy decision aids (including pamphlets) and newspaper articles are preferred and recommended for older adults (21, 40) but innovations to digitalize and/or gamify these materials and place at social locations should be considered to reach the tech-savvy and highly social younger adults.

At the interpersonal level, first and foremost, the investment in building trusting relationships between patients and doctors will also value-add and is well known to make medical consultations more effective. The cyclical interdependency between continuity of care and trust is key to enabling SDM processes and must not be underestimated. The practices of enrolling with one primary care doctor should be recommended at the national level and patients making annual check-in visits (especially for older adults) or having tele-consultations with a regular doctor (particularly for younger adults) should be encouraged. These touch points should be harnessed to distribute decision aids or message cards with focused and consistent evidence-based messages on appropriate antibiotic use and AMR. These can also be made available on “café cards” or posters through identified community networks with messages crafted to spark discussion, social engagement and awareness of AMR, and the potential effects of this on current and future generations.

In fact, the strategy of engaging healthcare professionals as educators and SDM facilitators was well supported. As highlighted in a recent systematic review conducted to assess the role of education in antibiotic stewardship (41), the distribution of passive educational materials to educate patients on antibiotic use without the presence of an active educator yielded negligible effects on improving antibiotic prescribing in the primary care setting (42–45). In contrast, the active involvement of adult patients and parents in SDM processes, through the use of a visual tool to clarify their values and preferences on antibiotics *via* conversations with their doctors, resulted in a 25% reduction of unnecessary antibiotics prescribed for upper respiratory tract infections (46). Educational tools were found to be impactful only when used as SDM decision aids; public engagement and education were also recommended to involve both doctors and community partners (41).

Such a strategy should aim to consciously streamline and design information flow, appealing to both younger and older generations, and especially drawing in younger adults. Younger adults are known to passively gather a variety of information *via* personal and friendship networks (47–50), including the Internet. Campaigns focused on inter-generational benefits and encouraging sharing about how to avoid AMR will increase the diffusion of messages and effectiveness of campaigns.

Coincidentally, our recommended strategic model aligns with a recent national healthcare reform in the community to build a healthier population in Singapore. From 2023 onwards, enrolment to a single preferred primary care provider will commence under the “Healthier SG” initiative to encourage continuity of care to address different health needs of Singapore residents at different stages of life, with the involvement of multiple care and community partners to promote healthy living for different age sub-populations (51). Riding on this initiative, it would spare the arduous process of lobbying for policy change prior to implementing our strategy. Instead, our implementation model can be applied immediately to the Singaporean context, leveraging on the affirmative infrastructure which will be established through this upcoming healthcare reform.

Our study had several strengths. Firstly, the use of mixed methods provided in-depth qualitative understanding on the complexities surrounding the community context and constructs known to be of interest in persuading patients to take antibiotics appropriately, namely, trust building, continuity of care, and SDM (18). Furthermore, for the survey component, we employed a robust sampling method to proportionately stratify and randomize accordingly, ensuring generalizability of quantitative results. Purposive, maximum variation sampling by age and ethnicity was used for the FGDs, to ensure a range of voices were captured for in-depth analysis, enabling transferability of findings. However, there was a low representation of participants aged 35–49 years from the Malay ethnic group.

Furthermore, we acknowledge the possibility of social desirability bias, which may have led study respondents to reduce their sharing of inappropriate antibiotic practices, though steps were taken to encourage open sharing, and emphasis placed on anonymity throughout data collection procedures. Finally, there could also be unknown confounders which were not adjusted for in the final logistic regression model.

## Conclusion

The current study demonstrates how building trust with a consistent provider opens up opportunities to educate the community on appropriate antibiotic use and AMR. The use of focused and consistent messaging in the community, the enablement of continuity of care with a regular primary care doctor, and leverage on the cultural capital of valuing SDM, to protect current and future generations from the fallout of AMR, is emphasized.

## Data availability statement

The original contributions presented in the study are included in the article/Supplementary material, further inquiries can be directed to the corresponding author/s.

## Ethics statement

The studies involving human participants were reviewed and approved by Domain Specific Review Board, National Healthcare Group, Singapore. The patients/participants provided their written informed consent to participate in this study.

## Author contributions

HG designed the FGD topic guide, co-designed the survey questionnaire, arranged and conducted training with the surveyors, analyzed the data, and drafted the manuscript. ZH provided guidance on data analysis and critically revised the manuscript. AC conceived the study, provided overall direction and planning for the study, co-designed the survey questionnaire, and critically revised the manuscript. All authors contributed to the article and approved the submitted version.

## Funding

This work was supported by the National Medical Research Council Singapore, Health Services Research Grant (NMRC/HSRG/0083/2017).

## Conflict of interest

The authors declare that the research was conducted in the absence of any commercial or financial relationships that could be construed as a potential conflict of interest.

## Publisher's note

All claims expressed in this article are solely those of the authors and do not necessarily represent those of their affiliated organizations, or those of the publisher, the editors and the reviewers. Any product that may be evaluated in this article, or claim that may be made by its manufacturer, is not guaranteed or endorsed by the publisher.

## Abbreviations

AMR: Antimicrobial resistance
COVID-19: Coronavirus disease 2019
FGD: Focus group discussion
GP: General practitioner
SDM: Shared decision-making
SRQR: Standards for Reporting Qualitative Research.

## References

[1] Review on Antimicrobial Resistance. Antimicrobial Resistance: Tackling a Crisis for the Health Wealth of Nations. (2014). Available online at: https://amr-review.org/sites/default/files/AMR%20Review%20Paper%20-%20Tackling%20a%20crisis%20for%20the%20health%20and%20wealth%20of%20nations_1.pdf (accessed January 21, 2022).

[2] World Health Organization. 14.9 Million Excess Deaths Associated With the COVID-19 Pandemic in 2020 and 2021 (2022). Available online at: https://www.who.int/news/item/05-05-2022-14.9-million-excess-deaths-were-associated-with-the-covid-19-pandemic-in-2020-and-2021 (accessed May 6, 2022).

[3] World Health Organization. Antibiotic Resistance. (2020). Available online at: https://www.who.int/news-room/fact-sheets/detail/antibiotic-resistance (accessed January 21, 2022).

[4] Zetts RM Stoesz A Garcia AM Doctor JN Gerber JS Linder JA. Primary care physicians' attitudes and perceptions toward antibiotic resistance and outpatient antibiotic stewardship in the USA: a qualitative study. BMJ Open. (2020) 10:e034983. 10.1136/bmjopen-2019-034983 32665343 PMC7365421

[5] Rose J Crosbie M Stewart A A. qualitative literature review exploring the drivers influencing antibiotic over-prescribing by GPs in primary care and recommendations to reduce unnecessary prescribing. Perspect Public Health. (2021) 141:19–27. 10.1177/1757913919879183 31633458

[6] O'Connor R O'Doherty J O'Regan A Dunne C. Antibiotic use for acute respiratory tract infections (ARTI) in primary care; what factors affect prescribing and why is it important? a narrative review. Ir J Med Sci. (2018) 187:969–86. 10.1007/s11845-018-1774-5 29532292 PMC6209023

[7] Germeni E Frost J Garside R Rogers M Valderas JM Britten N. Antibiotic prescribing for acute respiratory tract infections in primary care: an updated and expanded meta-ethnography. Br J Gen Pract. (2018) 68:e633–45. 10.3399/bjgp18X697889 29914880 PMC6104881

[8] Lee TH Wong JG Lye DC Chen MI Loh VW Leo YS. Medical and psychosocial factors associated with antibiotic prescribing in primary care: survey questionnaire and factor analysis. Br J Gen Pract. (2017) 67:e168–77. 10.3399/bjgp17X688885 28093423 PMC5325658

[9] Patel A Pfoh ER Misra Hebert AD Chaitoff A Shapiro A Gupta N. Attitudes of high vs. low antibiotic prescribers in the management of upper respiratory tract infections: a mixed methods study. J Gen Intern Med. (2020) 35:1182–8. 10.1007/s11606-019-05433-5 31630364 PMC7174444

[10] Yates TD Davis ME Taylor YJ Davidson L Connor CD Buehler K. Not a magic pill: a qualitative exploration of provider perspectives on antibiotic prescribing in the outpatient setting. BMC Fam Pract. (2018) 19:96. 10.1186/s12875-018-0788-4 29933762 PMC6015451

[11] Tonkin-Crine SK Tan PS van Hecke O Wang K Roberts NW McCullough A. Clinician-targeted interventions to influence antibiotic prescribing behavior for acute respiratory infections in primary care: an overview of systematic reviews. Cochrane Database Syst Rev. (2017) 9:CD012252. 10.1002/14651858.CD012252.pub2 28881002 PMC6483738

[12] Price L Gozdzielewska L Young M Smith F MacDonald J McParland J. Effectiveness of interventions to improve the public's antimicrobial resistance awareness and behaviors associated with prudent use of antimicrobials: A systematic review. J Antimicrob Chemother. (2018) 73:1464–78. 10.1093/jac/dky076 29554263

[13] Lim JM Singh SR Duong MC Legido-Quigley H Hsu LY Tam CC. Impact of national interventions to promote responsible antibiotic use: a systematic review. J Antimicrob Chemother. (2020) 75:14–29. 10.1093/jac/dkz348 31834401 PMC6910191

[14] Coxeter P Del Mar CB McGregor L Beller EM Hoffmann TC. Interventions to facilitate shared decision making to address antibiotic use for acute respiratory infections in primary care. Cochrane Database Syst Rev. (2015) 2015:CD010907. 10.1002/14651858.CD010907 26560888 PMC6464273

[15] Blyer K Hulton L. College students, shared decision making, and the appropriate use of antibiotics for respiratory tract infections: a systematic literature review. J Am Coll Health. (2016) 64:334–41. 10.1080/07448481.2015.1099105 26700137

[16] Brookes-Howell L Wood F Verheij T Prout H Cooper L Hood K. Trust, openness and continuity of care influence acceptance of antibiotics for children with respiratory tract infections: a four country qualitative study. Fam Pract. (2014) 31:102–10. 10.1093/fampra/cmt052 24165374

[17] Barrera SC Cancino RS Barreto TW. The impact of continuity of care on antibiotic prescribing in acute otitis media. Int J Pediatr Otorhinolaryngol. (2019) 126:109616. 10.1016/j.ijporl.2019.109616 31376791

[18] Guo H Hildon ZJ Loh VWK Sundram M Ibrahim MAB Tang WE. Exploring antibiotic prescribing in public and private primary care settings in Singapore: a qualitative analysis informing theory and evidence-based planning for value-driven intervention design. BMC Fam Pract. (2021) 22:205. 10.1186/s12875-021-01556-z 34654368 PMC8519324

[19] Nancy S Dongre AR. Behavior change communication: past, present, and future. Indian J Community Med. (2021) 46:186–90. 10.4103/ijcm.IJCM_441_20 34321723 PMC8281832

[20] Elwyn G Frosch D Thomson R Joseph-Williams N Lloyd A Kinnersley P. Shared decision making: a model for clinical practice. J Gen Intern Med. (2012) 27:1361–7. 10.1007/s11606-012-2077-6 22618581 PMC3445676

[21] Guo H Hildon ZJ Lye DCB Straughan P Chow A. The associations between poor antibiotic and antimicrobial resistance knowledge and inappropriate antibiotic use in the general population are modified by age. Antibiotics (Basel). (2022) 11:47. 10.3390/antibiotics11010047 35052924 PMC8773329

[22] O'Brien BC Harris IB Beckman TJ Reed DA Cook DA. Standards for reporting qualitative research: a synthesis of recommendations. Acad Med. (2014) 89:1245–51. 10.1097/ACM.0000000000000388 24979285

[23] Health Promotion Board. Programmes: Antibiotics do Not Treat Flu. (2021). Available online at: https://healthhub.sg/programmes/146/use-antibiotics-right (accessed January 21, 2022).

[24] Center for Health Protection Department of Health. General Public's Knowledge, Attitude and Practice Survey on Antimicrobial Resistance 2016/17. (2017).

[25] Hall MA Camacho F Dugan E Balkrishnan R. Trust in the medical profession: conceptual and measurement issues. Health Serv Res. (2002) 37:1419–39. 10.1111/1475-6773.01070 12479504 PMC1464022

[26] Cals JW Boumans D Lardinois RJ Gonzales R Hopstaken RM Butler CC. Public beliefs on antibiotics and respiratory tract infections: an internet-based questionnaire study. Br J Gen Pract. (2007) 57:942–7. 10.3399/096016407782605027 18252068 PMC2084132

[27] Hawkings NJ Butler CC Wood F. Antibiotics in the community: a typology of user behaviors. Patient Educ Couns. (2008) 73:146–52. 10.1016/j.pec.2008.05.025 18640805

[28] Brooks L Shaw A Sharp D Hay AD. Toward a better understanding of patients' perspectives of antibiotic resistance and MRSA: a qualitative study. Fam Pract. (2008) 25:341–8. 10.1093/fampra/cmn037 18647956

[29] Brookes-Howell L Elwyn G Hood K Wood F Cooper L Goossens H. 'The body gets used to them': patients' interpretations of antibiotic resistance and the implications for containment strategies. J Gen Intern Med. (2012) 27:766–72. 10.1007/s11606-011-1916-1 22065334 PMC3378752

[30] Hoffmann K Ristl R Heschl L Stelzer D Maier M. Antibiotics and their effects: what do patients know and what is their source of information? Eur J Public Health. (2014) 24:502–7. 10.1093/eurpub/ckt112 23960097

[31] Gualano MR Gili R Scaioli G Bert F Siliquini R. General population's knowledge and attitudes about antibiotics: a systematic review and meta-analysis. Pharmacoepidemiol Drug Saf. (2015) 24:2–10. 10.1002/pds.3716 25251203

[32] Pan DS Huang JH Lee MH Yu Y Chen MI Goh EH. Knowledge, attitudes and practices toward antibiotic use in upper respiratory tract infections among patients seeking primary health care in Singapore. BMC Fam Pract. (2016) 17:148. 10.1186/s12875-016-0547-3 27809770 PMC5094024

[33] Hennink MM Kaiser BN Weber MB. What influences saturation? estimating sample sizes in focus group research. Qual Health Res. (2019) 29:1483–96. 10.1177/1049732318821692 30628545 PMC6635912

[34] Guest G MacQueen KM Namey EE. Applied Thematic Analysis (2012).

[35] Department of Statistics Singapore. Singapore Census of Population (2020).

[36] Choo SJ Chang CT Lee JCY Munisamy V Tan CK Raj JD. A cross-sectional study on public belief, knowledge and practice toward antibiotic use in the state of Perak, Malaysia. J Infect Dev Ctries. (2018) 12:960–9. 10.3855/jidc.10723 32012125

[37] Lim JM Chhoun P Tuot S Om C Krang S Ly S. Public knowledge, attitudes and practices surrounding antibiotic use and resistance in Cambodia. JAC Antimicrob Resist. (2021) 3:dlaa115. 10.1093/jacamr/dlaa115 34223067 PMC8210153

[38] Gillani AH Chang J Aslam F Saeed A Shukar S Khanum F. Public knowledge, attitude, and practice regarding antibiotics use in Punjab, Pakistan: a cross-sectional study. Expert Rev Anti Infect Ther. (2021) 19:399–411. 10.1080/14787210.2021.1823216 32912015

[39] World Health Organization. Effective Communications: Participant Handbook: Communications Training Programme for WHO Staff. London: Geneva (2015).

[40] Guo H Lim HY Chow A. Health information orientation profiles and their association with knowledge of antibiotic use in a population with good Internet access: a cross-sectional study. Antibiotics. (2022) 11:769. 10.3390/antibiotics11060769 35740175 PMC9220153

[41] Satterfield J Miesner AR Percival KM. The role of education in antimicrobial stewardship. J Hosp Infect. (2020) 105:130–41. 10.1016/j.jhin.2020.03.028 32243953

[42] Gonzales R Sauaia A Corbett KK Maselli JH Erbacher K Leeman-Castillo BA. Antibiotic treatment of acute respiratory tract infections in the elderly: effect of a multidimensional educational intervention. J Am Geriatr Soc. (2004) 52:39–45. 10.1111/j.1532-5415.2004.52008.x 14687313

[43] Mainous AG Diaz VA Carnemolla M. A community intervention to decrease antibiotics used for self-medication among Latino adults. Ann Fam Med. (2009) 7:520–6. 10.1370/afm.1061 19901311 PMC2775608

[44] Smeets HM Kuyvenhoven MM Akkerman AE Welschen I Schouten GP van Essen GA. Intervention with educational outreach at large scale to reduce antibiotics for respiratory tract infections: a controlled before and after study. Fam Pract. (2009) 26:183–7. 10.1093/fampra/cmp008 19258441

[45] Vinnard C Linkin DR Localio AR Leonard CE Teal VL Fishman NO. Effectiveness of interventions in reducing antibiotic use for upper respiratory infections in ambulatory care practices. Popul Health Manag. (2013) 16:22–7. 10.1089/pop.2012.0025 23113630 PMC3595097

[46] Légaré F Labrecque M Cauchon M Castel J Turcotte S Grimshaw J. Training family physicians in shared decision-making to reduce the overuse of antibiotics in acute respiratory infections: a cluster randomized trial. CMAJ. (2012) 184:E726–34. 10.1503/cmaj.120568 22847969 PMC3447039

[47] Wrzus C Hänel M Wagner J Neyer FJ. Social network changes and life events across the life span: a meta-analysis. Psychol Bull. (2013) 139:53–80. 10.1037/a0028601 22642230

[48] Carstensen LL. Socioemotional selectivity theory: Social activity in life-span context. Annu Rev Gerontol Geriatr. (1991) 11:195–217.

[49] Carstensen LL. Social and emotional patterns in adulthood: Support for socioemotional selectivity theory. Psychol Aging. (1992) 7:331–8. 10.1037/0882-7974.7.3.331 1388852

[50] Carstensen LL. Evidence for a life-span theory of socioemotional selectivity. Curr Dir Psychol Sci. (1995) 4:151–6. 10.1111/1467-8721.ep1151226127213487

[51] Ministry of Health Singapore. News Highlight: Promoting Overall Healthier Living While Targeting Specific Sub-Populations. (2022). Available online at: https://www.moh.gov.sg/news-highlights/details/promoting-overall-healthier-living-while-targeting-specific-sub-populations (accessed September 6, 2022).

