# Supplementary material for: “Antibiotics are for everyone, our past and our future generations, right? If antibiotics are dead, we will be in big trouble”: Building on community values for public engagement on appropriate use of antibiotics in Singapore
Source: Front Public Health. 2022 Sep 30;10:1001282. doi: 10.3389/fpubh.2022.1001282 (PMC9561345; doi:10.3389/fpubh.2022.1001282)
Supplement: Supplementary file 1 [file Data_Sheet_1.docx]

**Perception and knowledge of antibiotic use and antibiotic resistance among the general population of Singapore**

Focus Group Discussion Guide

Objectives:

1. To ascertain the awareness, perception and experience of antibiotic use among the general public in the community
2. To ascertain the awareness, perception and experience of antibiotic resistance among the general public in the community

FGD Questions:

1. In the event where you are down with an acute condition (such as cough, sore throat or runny nose), what is the first thing that you would do to manage your symptoms?
2. In Singapore, we are shaped by our traditional roots in our daily living. What influences how you seek health when you are sick? How does it affect you in the need to seek immediate care or treatment for an acute condition?
3. Please tell me what you know about antibiotics.
4. How many of you have used antibiotics before?
   1. [If Yes, ask:] Would you mind sharing your experience of using antibiotics for e.g. why were you prescribed, how do you feel after taking the antibiotics?
   2. [If No, ask:] Have you heard from any of your family or friends on their experience of using antibiotics? Would you mind sharing their experiences?
5. Where did you get your antibiotics from?
6. Would you describe some situations when you have requested for antibiotics from your doctor and why did you do so?
7. Were there situations where you were prescribed with antibiotics by your doctor and you rejected it? Please elaborate.
8. A short scenario for all. The doctor prescribes a course of antibiotic for you. After taking it for 2-3 days you start feeling better. Do you…
   1. Stop taking the antibiotics
   2. Save the remaining antibiotics for the next time you get sick
   3. Discard the remaining antibiotics
   4. Finish the course of the antibiotics
   5. Give the remaining antibiotics to your family/friends the next time they fall sick

What are the reasons for choosing the following? Could you elaborate more?

1. Would you describe some situations when you have heard of friends or family members requesting, or rejecting antibiotics, sharing antibiotics, keeping antibiotics for future use or stopping antibiotics early? What’s your opinion of these practices?
2. Has anyone heard of “antibiotic resistance” prior to this discussion?
   1. [If No, ask:] When you hear the phrase “antibiotic resistance”, would you describe what comes to your mind?
   2. [If Yes, ask:] Where did you hear about it from and how would you describe antibiotic resistance?
3. Which of the following do you think contributes to antibiotic resistance? You may select more than one option. (Show of hands for each option):
   1. Not finishing a course of Antibiotics
   2. Getting Antibiotics for viral infection
   3. Using strong antibiotics for mild bacterial infection

Have you used any of these practices before? Please elaborate.

1. How serious do you perceived the severity of antibiotic resistance in Singapore? Is it High, Low and Not sure? Why?
2. Which of the following do you think will be affected by antibiotic resistance?
   1. Individual (you)
   2. Elderly
   3. Children
   4. Hospital patients
   5. Community

Out of all this choices, who do you think will be at higher risk of developing antibiotic resistance? Why do you say so?

1. Can you tell me what do you think is your chances of being resistant to antibiotics? Is it High, Low or Not Sure?
2. Who do you think may be responsible in reducing antibiotic resistance?
3. Do you think that you have a role to play in terms of protecting yourself against antibiotic resistance?
   1. How can you protect yourself from antibiotic resistance?
4. How would you describe the current knowledge and perceptions of the public have in terms of antibiotics use and antibiotics resistance when seeking clinical care?
   1. Where do you think the problem lies and how can we further improve antibiotic use?

Supplementary Table S1: Socio-demographic characteristics of Singapore residents surveyed between November 2020 and January 2021, N=2004, compared to Census 2020.

| **Socio-demographics compared to Census 2020** | **Survey respondents,**  **%** | **Singapore residents in Census 2020^a^,**  **%** |
| --- | --- | --- |
| ***Residency status*** |  |  |
| Singapore citizen | 87 | 86 |
| Permanent resident | 13 | 14 |
| ***Gender*** |  |  |
| Female | 52 | 52 |
| ***Ethnicity*** |  |  |
| Chinese | 72 | 76 |
| Malay | 15 | 13 |
| Indian | 11 | 8 |
| Others | 3 | 3 |
| ***Age group*** |  |  |
| 21-34 years old | 31 | 26 |
| 35-49 years old | 33 | 28 |
| ≥50 years old | 36 | 46 |
| ***Highest education level*** |  |  |
| Lower educated (post-secondary & below) | 35 | 51 |
| Higher educated (diploma & above) | 65 | 49 |
| ***Housing type^b^*** |  |  |
| HDB 1- and 2-room flats | 6 | 6 |
| HDB 3-room flats | 20 | 18 |
| HDB 4-room flats | 35 | 32 |
| HDB 5-room and executive flats | 20 | 23 |
| Condominiums and other apartments | 14 | 16 |
| Landed properties | 5 | 5 |

*^a^ Census data includes population who are 20 years and above;*

*^b^ HDB (or Housing Development Board) flats refer to public housing provided by Singapore and the number of rooms is a surrogate marker of a household’s socioeconomic status*

Supplementary Table S2: Health and antibiotic related characteristics Singapore residents surveyed between November 2020 and January 2021, N=2004.

| **Health and antibiotic-related characteristics** | **Survey respondents,**  **%** |
| --- | --- |
| ***Family/Friend working in healthcare sector*** |  |
| Yes | 54 |
| ***Seek medical attention from same regular doctor?*** |  |
| Yes | 62 |
| ***Reported to have at least one chronic illness*** |  |
| Yes | 32 |
| ***When was antibiotics last taken?*** |  |
| In the last month | 6 |
| In the last 6 months | 16 |
| In the last year | 17 |
| More than a year ago | 36 |
| Cannot remember | 21 |
| Never | 3 |
| ***Source of antibiotics (on occasion antibiotics last taken)^a^*** |  |
| General Practitioner (GP) clinic in Singapore^b^ | 66 |
| Polyclinic in Singapore^c^ | 17 |
| Hospital in Singapore | 10 |
| Overseas | 1 |
| The internet | 0 |
| Friends or family members | 0.2 |
| Antibiotics saved up from previous time | 1 |
| Cannot remember | 6 |
| ***Ever received advice from a doctor, nurse or pharmacist on how to take the antibiotics*** ***(on occasion antibiotics last taken)^a^*** |  |
| Yes | 92 |
| ***Completed prescribed antibiotics course (on occasion antibiotics last taken)^a^*** |  |
| Yes | 81 |

*^a^Of 1948 respondents who had ever taken antibiotics in their lifetime;*

*^b^Privately-funded primary care clinic in Singapore;*

*^c^Government-funded primary care clinic in Singapore.*

Supplementary Table S3: Demographics of 91 participants amongst 13 FGDs conducted between August 2018 and September 2020.

| **Demographics** | **Chinese** | **Malay** | **Indian** | **Overall** |
| --- | --- | --- | --- | --- |
| ***Number of FGDs*** | 5 | 4 | 4 | 13 |
| ***Number of participants*** | 36 | 28 | 27 | 91 |
| ***Gender*** | | | | |
| Female | 25 (69) | 17 (61) | 21 (78) | 63 (69) |
| Male | 11 (31) | 11 (39) | 6 (22) | 28 (31) |
| ***Age group*** | | | | |
| 21-34 years old | 8 (22) | 13 (46) | 6 (22) | 27 (30) |
| 35-49 years old | 4 (11) | 1 (4) | 8 (30) | 13 (14) |
| ≥50 years old | 24 (67) | 14 (50) | 13 (48) | 51 (56) |
| ***Highest education Level*** | | | | |
| Lower educated (Post-secondary & below) | 15 (42) | 18 (64) | 11 (41) | 44 (48) |
| Higher educated (Diploma & above) | 21 (58) | 10 (36) | 16 (59) | 47 (52) |

Supplementary Table S4: Suggested message content based on study findings.

| 1. Antibiotics are used to treat bacterial infections, such as urinary tract infections, skin infections and tuberculosis. They do not treat viral infections such as COVID-19 and common cold or flu. |
| --- |
| 1. Antibiotics do not fight pain nor have anti-inflammatory properties; you will need something else for this, like ibuprofen or aspirin. |
| 1. Antibiotic resistance occurs when the bacteria (not the body) defeat the antibiotics that were designed to kill them. |
| 1. Unnecessary or overuse of antibiotics can cause bacteria to develop resistance. This is termed as antibiotic resistance. Antibiotic resistance will compromise present and future generations’ ability to fight bacterial infections. |
| 1. Not finishing a course of antibiotics as advised encourages the development of antibiotic-resistant bacteria. |
| 1. Antibiotic-resistant infections can happen to anyone, leading to longer recovery periods and more expensive and intensive treatments. |
| 1. Not all illnesses are the same, even if you have the same symptoms. Self-diagnosis and inappropriate self-medication with antibiotics can lead to antibiotic resistance. |
| 1. Sharing antibiotics without a doctor’s prescription is not advisable. One should always complete the prescribed course of antibiotic treatment and avoid self-medication using left-over antibiotics to prevent antibiotic resistance. |
| 1. Always seek consultation with a doctor and agree on the best course of treatment before deciding to take antibiotics. |
| 1. Recovery will be faster and safer on the right antibiotic course, taken as advised and prescribed. |
